# Differential Effects of Histidine and Histidinamide versus Cysteine and Cysteinamide on Copper Ion-Induced Oxidative Stress and Cytotoxicity in HaCaT Keratinocytes

**DOI:** 10.3390/antiox12040801

**Published:** 2023-03-25

**Authors:** Jae Won Ha, Joon Yong Choi, Yong Chool Boo

**Affiliations:** 1 Department of Biomedical Science, The Graduate School, Kyungpook National University, 680 Gukchaebosang-ro, Jung-gu, Daegu 41944, Republic of Korea; jaewon1226@knu.ac.kr (J.W.H.); halo134679@knu.ac.kr (J.Y.C.); 2 BK21 Plus KNU Biomedical Convergence Program, Kyungpook National University, 680 Gukchaebosang-ro, Jung-gu, Daegu 41944, Republic of Korea; 3 Department of Molecular Medicine, School of Medicine, Kyungpook National University, 680 Gukchaebosang-ro, Jung-gu, Daegu 41944, Republic of Korea; 4 Cell and Matrix Research Institute, Kyungpook National University, 680 Gukchaebosang-ro, Jung-gu, Daegu 41944, Republic of Korea

**Keywords:** copper ion, CuSO_4_, chelator, amino acid, histidine, histidinamide, cysteine, cysteinamide, HaCaT cells, viability, oxidative stress

## Abstract

Metal chelators are used for various industrial and medical purposes based on their physicochemical properties and biological activities. In biological systems, copper ions bind to certain enzymes as cofactors to confer catalytic activity or bind to specific proteins for safe storage and transport. However, unbound free copper ions can catalyze the production of reactive oxygen species (ROS), causing oxidative stress and cell death. The present study aims to identify amino acids with copper chelation activities that might mitigate oxidative stress and toxicity in skin cells exposed to copper ions. A total of 20 free amino acids and 20 amidated amino acids were compared for their copper chelation activities in vitro and the cytoprotective effects in cultured HaCaT keratinocytes exposed to CuSO_4_. Among the free amino acids, cysteine showed the highest copper chelation activity, followed by histidine and glutamic acid. Among the amidated amino acids, cysteinamide showed the highest copper chelation activity, followed by histidinamide and aspartic acid. CuSO_4_ (0.4–1.0 mM) caused cell death in a concentration-dependent manner. Among the free and amidated amino acids (1.0 mM), only histidine and histidinamide prevented the HaCaT cell death induced by CuSO_4_ (1.0 mM). Cysteine and cysteinamide had no cytoprotective effects despite their potent copper-chelating activities. EDTA and GHK-Cu, which were used as reference compounds, had no cytoprotective effects either. Histidine and histidinamide suppressed the CuSO_4_-induced ROS production, glutathione oxidation, lipid peroxidation, and protein carbonylation in HaCaT cells, whereas cysteine and cysteinamide had no such effects. Bovine serum albumin (BSA) showed copper-chelating activity at 0.5–1.0 mM (34–68 mg mL^−1^). Histidine, histidinamide, and BSA at 0.5–1.0 mM enhanced the viability of cells exposed to CuCl_2_ or CuSO_4_ (0.5 mM or 1.0 mM) whereas cysteine and cysteinamide had no such effects. The results of this study suggest that histidine and histidinamide have more advantageous properties than cysteine and cysteinamide in terms of alleviating copper ion-induced toxic effects in the skin.

## 1. Introduction

Metal chelators refer to compounds with physicochemical properties that trap and bind specific metal ions. Different types of metal chelators are used in various industrial fields for the management of resources and wastes [1,2]. Numerous chelators are also used in medicine to remove excess metals from the body or restore disturbed homeostasis of essential metals [3]. Well-designed chelators are used in the synthesis of thermodynamically and kinetically stable complexes with radioactive isotopes of metal ions for nuclear medicine applications [4]. Copper and iron chelating agents are used in food, cosmetics, and pharmaceuticals for the regulation of metal metabolism and the redox biology of cells [5,6].

Copper is a trace metal element essential for normal human metabolism [7]. Copper is an essential cofactor for several metalloproteins, such as cytochrome C oxidase and superoxide dismutase [8]. Copper deficiency is often recognized clinically as anemia, leucopenia, and myeloneuropathy [9]. Copper toxicity can result from exposure to high levels of copper from contaminated food, water sources, and air in the areas of copper smelters and mines [10]. Medical conditions that limit the hepatic removal of excess copper from the body, or certain genetic disorders, such as Menke’s disease and Wilson’s disease, can lead to copper toxicity [11]. Excess copper can cause diarrhea, headaches, hepatic disorder, kidney failure, and neurodegenerative changes [12].

The majority of serum copper is transported bound to ceruloplasmin, albumin, transcuprein, and amino acids, keeping the concentration of free copper ions very low [13]. Liberated free copper ions can catalyze Fenton-type reactions to produce reactive oxygen species (ROS) [14]. The cytotoxic effects of copper ions are variable depending on their salt forms and cell types. The toxic effects of copper chloride (CuCl_2_) and copper acetate (Cu(OAc)_2_ on HaCaT cell viability and irritation markers appear to be greater than those of copper tripeptide (GHK-Cu) [15]. Copper oxide (CuO) is more toxic than copper sulfate (CuSO_4_) and astrocytoma and glioblastoma cells are more vulnerable to copper toxicity than neuronal cells [16]. CuCl_2_ enhances the production of ROS and reactive nitrogen species (RNS), protein oxidation, lipid peroxidation, and DNA damage while decreasing glutathione (GSH), total sulphydryl content, and the activities of many antioxidant enzymes in human erythrocytes [17]. CuSO_4_ also induces oxidative stress leading to mitochondrial dysfunction, apoptosis, and autophagy in immortalized male germ cell line GC-1 [18]. Different molecules with copper chelation properties are already in clinical use or under clinical trial to treat neurodegenerative diseases, cardiovascular disorders, pulmonary fibrosis, diabetes, and cancers [5,19].

Free amino acids including histidine bind to copper with a high affinity and form stable complexes [20]. The copper–histidine complex found in serum contributes to the transportation of copper from albumin into cells [21]. The copper–histidine complex provides a copper replacement for patients with Menke’s disease, supplying copper directly by injection into the body, bypassing intestinal routes [22]. Histidine inhibited the copper (II)-neocuproine complex-catalyzed autoxidation of ascorbic acid most effectively among the tested amino acids [23]. Supplementation of histidine in fish diet prevented CuSO_4_-induced oxidative damage in the intestine of grass carp (*Ctenopharyngodon idella*) [24]. Thus, it is expected that amino acids can have variable effects against copper-induced toxic effects. However, there has been no study that has directly compared and evaluated the antioxidant action of various amino acids against the toxicity of copper ions in cells.

Chelators can act as either antioxidants or prooxidants by inhibiting or enhancing the transition metal-catalyzed production of ROS [25]. Thus, it is hypothesized that only certain types of copper chelators may have beneficial effects in cells exposed to high levels of copper ions. The goal of the present study is to compare and identify amino acids that effectively chelate copper ions and mitigate the oxidative stress induced by copper ions in skin cells. For this purpose, the copper chelation activities of a total of 20 free amino acids and 20 amidated amino acids were comprehensively evaluated. The amidated amino acids were included in this study because several amidated amino acids have shown distinct biological properties compared to free amino acids in our previous studies [26,27,28]. As a result of primary screening, cysteine, cysteinamide, histidine, and histidinamide were shown to have potent copper-chelating activity. The effects of these four compounds on copper ion-induced cytotoxicity and oxidative stress were further explored in human HaCaT keratinocytes. The results showed for the first time that histidine and histidinamide, but not cysteine and cysteinamide, enhanced the viability of cells exposed to high concentrations of copper ions, and inhibited copper ion-induced ROS production, GSH oxidation, lipid peroxidation, and protein carbonylation, suggesting the former two compounds have more advantageous properties as copper chelators in terms of alleviating the toxic effects of copper ions.

## 2. Materials and Methods

### 2.1. Reagents

Naturally occurring free amino acids were purchased from Sigma-Aldrich (St. Louis, MO, USA) and amidated amino acids were purchased from Watanabe Chemical Ind., Ltd. (Hiroshima, Japan). CuCl_2_, CuSO_4_, glycyl-histidyl-lysine-copper (GHK-Cu), ethylenediamine-N,N,N’,N’-tetraacetic acid (EDTA), pyrocatechol violet (PCV), 2′,7′-dichlorodihydrofluorescein diacetate (DCFH-DA), 2-thiobarbituric acid (TBA), and 1,1,3,3-tetramethoxypropane (TMP) were purchased from Sigma-Aldrich (St. Louis, MO, USA). 3-(4,5-Dimethylthiazol-2-yl)-2,5-diphenyltetrazolium bromide (MTT) was purchased from Amresco (Solon, OH, USA). Bovine serum albumin (BSA) was purchased from GenDEPOT (Houston, TX, USA).

### 2.2. Assay of Copper-Chelating Activity

The copper-chelating activity was determined through a spectrophotometric assay using PCV [29]. In this assay, the Cu(II)-PCV complex shows maximum absorption at 632 nm, and the absorbance is reduced by a copper chelator competing with PCV for a fixed amount of copper. The reaction mixture containing 200 μM test material, 200 μM CuSO_4_, and 200 μM PCV in 50 mM sodium acetate buffer (pH 6.0) was incubated at 25 °C for 1 min or 20 min, and its absorbance at 632 nm (A_632_) was recorded using a Spectrostar Nano microplate reader (BMG LABTECH GmbH, Ortenberg, Germany).

### 2.3. Cell Culture

HaCaT cells (CLS Cell Lines Service GmbH, Eppelheim, Germany) were maintained under humidified air containing 5% CO_2_ in a closed incubator at 37 °C. Cells were fed with DMEM/F-12 medium (GIBCO-BRL, Grand Island, NY, USA) supplemented with 10% fetal bovine serum, 100 U·mL^−1^ penicillin, 100 µg·mL^−1^ streptomycin, and 0.25 µg·mL^−1^ amphotericin B.

### 2.4. Assay of Cell Viability

Cells were seeded onto 96-well culture plates (4 × 10^3^ cells per well), cultured in the growth medium (200 µL) for 24 h, and then treated with each test material in the absence or presence of CuSO_4_ or CuCl_2_ at the specified concentrations for an additional 48 h. Cell viability was assessed using the tetrazolium MTT dye, which is reduced to insoluble purple formazan by cellular oxidoreductases in live cells [30].

### 2.5. Assay of ROS Production

Flow cytometry was used to count cells emitting fluorescence due to the oxidation of DCFH-DA by ROS inside cells [31,32]. Cells were seeded on 6-well culture plates (2 × 10^5^ cells per well) and cultured in the growth medium (2 mL) for 3 h. The spent medium was replaced with the growth medium containing various test materials, followed by incubation of the cells for 3 h. After discarding the medium, the adherent cells were washed with PBS, detached from the culture plates using a trypsin/EDTA solution (200 µL), and collected in a microcentrifuge tube. After centrifugation of the tubes at 316× *g* for 3 min with a Combi 408 centrifuge (Hanil, Daejeon, Republic of Korea), the precipitated cells were washed with PBS, labeled with 10 µM DCFH-DA for 30 min, and suspended in PBS for flow cytometry. Flow cytometric analysis of the cell suspension was conducted using BD FACSCalibur (BD Biosciences, San Jose, CA, USA), followed by data analysis using BD CellQuest. Data are presented as the ratio (%) of cells with high fluorescence due to intracellular ROS production to the total gated cells.

### 2.6. Assay of GSH and Its Oxidized Form, Glutathione Disulfide (GSSG)

Cells were seeded on 6-well culture plates (2 × 10^5^ cells per well) and cultured in the growth medium (2 mL) for 24 h. After treatment with each test material for an additional 12 h, the spent medium was discarded, and the adherent cells were washed with PBS, extracted with 5% *meta*-phosphoric acid solution (150 µL per well), and centrifuged at 14,500× *g* for 15 min to obtain the supernatant. The contents of GSH and GSSG were measured using a GSH/GSSG assay kit (GT40) from Oxford Biomedical Research (Oxford, UK) [33,34,35]. GSH contents were normalized to the total protein contents.

### 2.7. Preparation of Whole-Cell Lysates and Protein Assay

Cells were seeded on 6-well culture plates (2 × 10^5^ cells per well) and cultured in the growth medium (2 mL) for 24 h. After treatment with each test material for an additional 12 h, the spent medium was discarded and the adherent cells were lysed with the lysis buffer A (20 mM Tris-Cl, 2.5 mM EDTA, 1.0% sodium dodecyl sulfate, pH 7.5, 150 µL per well). The protein content of the whole-cell lysates was determined using a DC protein assay kit (Bio-Rad, Hercules, CA, USA). The whole-cell lysates were used for the assays of lipid peroxidation and protein carbonylation.

### 2.8. Assay of Lipid Peroxidation

Lipid peroxidation was assessed using a TBA method [36]. Whole-cell lysate (60 μg protein in 100 μL) was mixed with 1.0% *meta*-phosphoric acid (50 μL) and 0.9% TBA (350 μL) in an air-tight microcentrifuge tube and heated in a boiling water bath for 45 min. After cooling, n-butyl alcohol (500 μL) was added to the tube, then it was vortex-mixed and centrifuged at 14,500× *g* for 15 min to separate the mixture into two layers. The upper layer (200 µL) was transferred to a black 96-well plate and its fluorescence intensity (excitation at 544 nm and emission at 590 nm) was measured using a Gemini EM fluorescence microplate reader (Molecular Devices, Sunnyvale, CA, USA). A standard curve was prepared using a TMP solution instead of the cell lysates. The data are presented as thiobarbituric acid-reactive substance (TBARS) content normalized to the protein content.

### 2.9. Assay of Protein Carbonylation

Protein carbonylation was measured using a protein carbonyl content fluorometric assay kit (ab235631) from Abcam (Cambridge, MA, USA). Whole-cell lysate (50 μg protein in 50 μL) was mixed with 50 µL of 200 μM fluorescein-5-thiosemicarbazide (FTC) fluorophore in an assay buffer in a microcentrifuge tube, followed by incubation overnight at 25 °C in the dark. Then, 200 µL of ice-cold 20% trichloroacetic acid solution was added and the tubes were left on ice for 10 min. After centrifugation of the tubes at 14,500× *g* for 15 min, the supernatant was discarded by aspiration. The pellet was washed with 200 µL of ice-cold isopropanol 3 times and air-dried. The pellet was dissolved in 50 µL of 6 M guanidine solution at 50 °C for 1 h. After cooling, the sample was mixed with 70 µL of sample dilution buffer. Aliquots of the diluted samples (100 µL) were transferred to a 96-well plate for fluorescence measurement at 485 nm excitation and 535 nm emission using a Gemini EM fluorescence microplate reader. Protein carbonyl contents were estimated by comparing a standard curve prepared using FTC fluorophore. Protein carbonyl contents were normalized to the protein content.

### 2.10. Statistical Analysis

Statistical analysis of experimental data was conducted using SigmaStat v.3.11 software (Systat Software Inc., San Jose, CA, USA). Data are presented as the mean ± standard deviation (SD) of multiple independent experiments. The presence of significantly different group means among all test groups was determined using a one-way analysis of variance (ANOVA) at *p* < 0.05 level. As a post hoc test, Duncan’s multiple range test was run to compare all groups to each other. The graph was created using Prism software version 6.0 (GraphPad Software, San Diego, CA, USA).

## 3. Results

### 3.1. Copper-Chelating Activities of Various Free and Amidated Amino Acids

**Figure 1:**
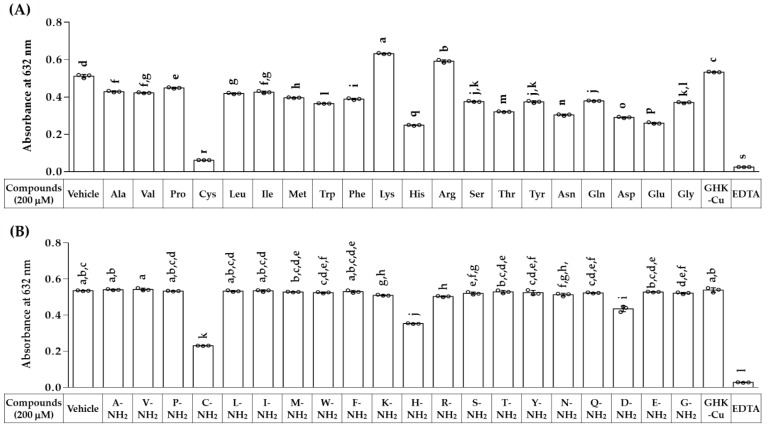
Copper-chelating activities of free amino acids (**A**) and amidated amino acids (**B**). The reaction mixture consisting of 200 μM CuSO_4_, 200 μM PCV, and test material at the specified concentrations was incubated for 20 min and the formation of Cu(II)-PCV complex was measured using the absorbance at 632 nm (A_632_). Ethylenediamine-N,N,N’,N’-tetraacetic acid (EDTA) and glycyl-histidyl-lysine-copper (GHK-Cu) were used for comparative purposes. Free amino acids are denoted using three-letter codes and amidated amino acids are denoted using one-letter codes with an amine group (NH_2_). Data represent mean ± SD (n = 3). Different lowercase letters (a–s) indicate significantly different means at the *p* < 0.05 level.

The formation of Cu(II)-PCV complex in the absence or presence of a free or amidated amino acid was measured to compare their copper-chelating properties. In this competitive assay, the reaction mixture contained a test material, CuSO_4_, and PCV, all at 200 μM in an aqueous solution. As shown in Figure 1, A_632_ was much reduced by EDTA, a metal chelator used as a positive control, compared to the vehicle control. GHK-Cu complex did not affect the A_632_ value, indicating that it did not affect the formation of Cu(II)-PCV complex. Most free amino acids except for lysine and asparagine reduced A_632_ due to the formation of Cu(II)-PCV complex. Cysteine reduced A_635_ most effectively, followed by histidine, glutamic acid, and aspartic acid. Of the free amino acids, cysteinamide, histidinamide, and aspartamide reduced A_635_ most effectively, in that order. The results suggest that cysteine, cysteinamide, histidine, and histidinamide have potent copper-chelating capacities.

### 3.2. CuSO_4_ Reduces Cell Viability While Increasing ROS Production of HaCaT Keratinocytes

**Figure 2:**
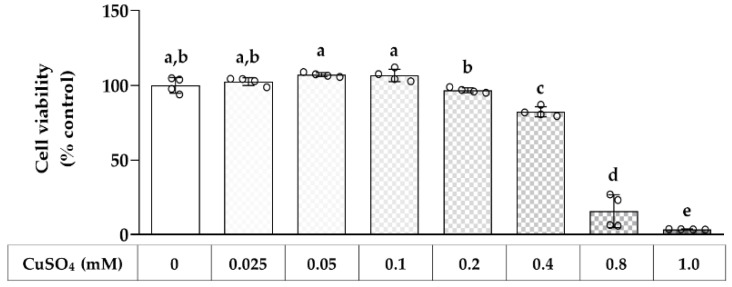
Effects of CuSO_4_ on the viability of HaCaT cells. Cells were treated with CuSO_4_ at varying concentrations for 48 h for the viability assay. Cell viability is presented as % of the control group (mean ± SD, n = 4). Different lowercase letters (a–e) indicate significantly different means at the *p* < 0.05 level.

To examine the cytotoxic effects of copper ions, human HaCaT keratinocytes were exposed to CuSO_4_ at different concentrations (0.25–1.0 mM) for 48 h, and cell viability was assessed. As shown in Figure 2, CuSO_4_ reduced the viability of HaCaT in a dose-dependent manner.

### 3.3. Effects of Free and Amidated Amino Acids on HaCaT Cell Viability in the Absence and Presence of CuSO_4_

**Figure 3:**
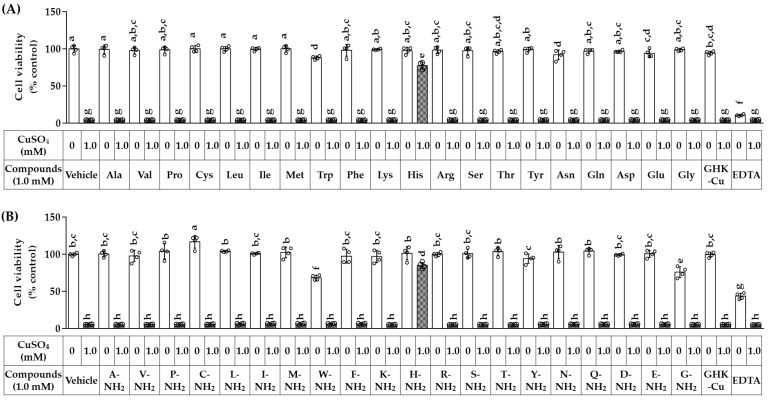
Effects of free amino acids (**A**) and amidated amino acids (**B**) on the viability of HaCaT cells in the absence and presence of CuSO_4_. Cells were treated with a test material at 1.0 mM with no or 1.0 mM CuSO_4_ for 48 h. GHK-Cu and EDTA were used as reference compounds. Cell viability was presented as % of the control group (mean ± SD, n = 4). Different lowercase letters (a–h) indicate significantly different means at the *p* < 0.05 level.

The effects of various free and amidated amino acids on cell viability were examined at 1.0 mM in the absence and presence of 1.0 mM CuSO_4_, and the results were compared to that of GHK-Cu or EDTA. As shown in Figure 3A, of the free amino acids tested, tryptophan and asparagine decreased cell viability a little, and others had no significant effects in the absence of CuSO_4_. Only histidine exhibited cytoprotective effects under CuSO_4_-treated conditions. As shown in Figure 3B, of the amidated amino acids tested, tryptophanamide and glycinamide decreased cell viability significantly, while cysteinamide increased it a little. Only histidinamide exhibited cytoprotective effects under CuSO_4_-treated conditions. GHK-Cu had no significant effects on cell viability in the absence of CuSO_4_, but EDTA itself showed severe cytotoxicity. GHK-Cu and most amino acids except for histidine and histidinamide failed to restore cell viability under CuSO_4_-treated conditions.

### 3.4. Dose-Dependent Effects of Histidine, Histidinamide, Cysteine, Cysteinamide, and EDTA on the CuSO_4_-Induced Death of HaCaT Cells

**Figure 4:**
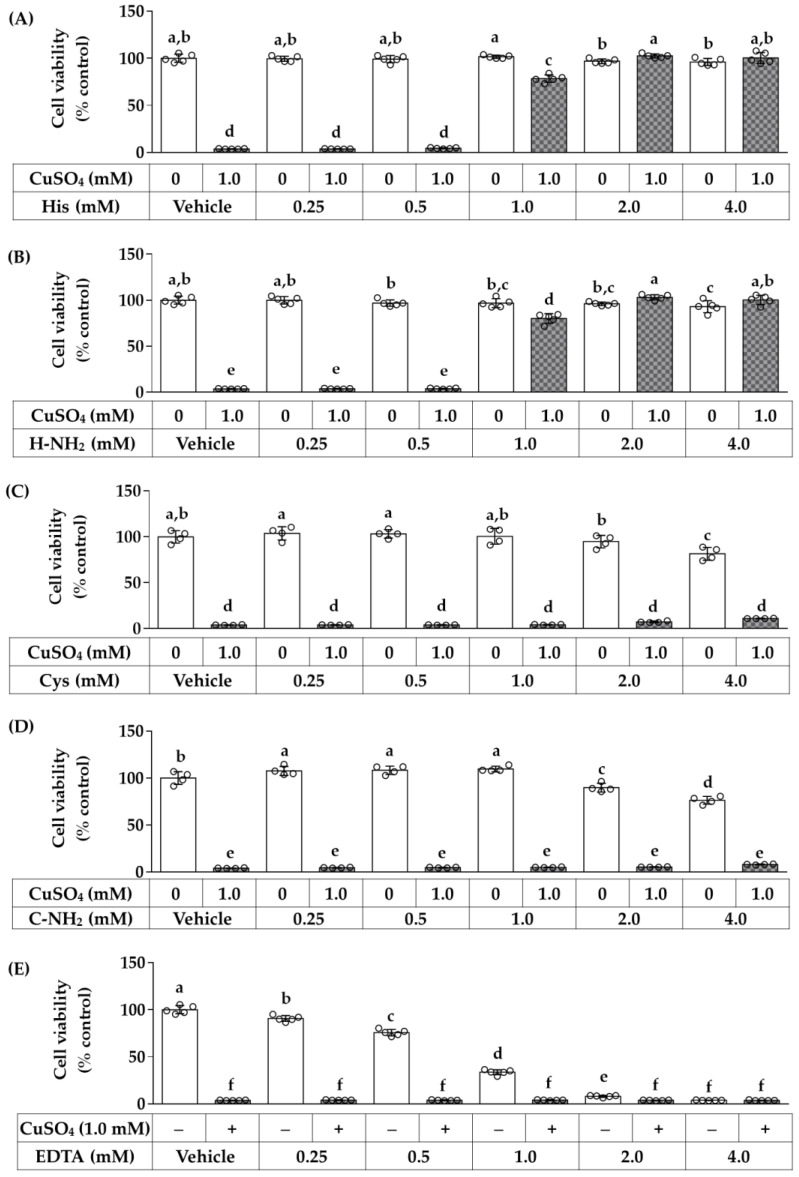
Effects of histidine (**A**), histidinamide (**B**), cysteine (**C**), cysteinamide (**D**), and EDTA (**E**) on the CuSO_4_-induced cytotoxicity. HaCaT cells were treated with a test material at varying concentrations in the absence and presence of 1.0 mM CuSO_4_ for 48 h. Cell viability was presented as % of the control group (mean ± SD, n = 4 or 5). Different lowercase letters (a–f) indicate significantly different means at the *p* < 0.05 level.

As above, although cysteine and cysteinamide showed stronger copper chelation activities than histidine and histidinamide, the latter two compounds alleviated the CuSO_4_-induced cytotoxicity more effectively than the former two compounds. Their effects on cell viability were additionally compared with EDTA at varying concentrations (0.25–1.0 mM) in the absence and presence of CuSO_4_ (1.0 mM). As shown in Figure 4, histidine and histidinamide rescued the cells exposed to CuSO_4_ (1.0 mM) at 1.0 mM and higher concentrations. They did not show cytotoxic effects up to 4.0 mM. In contrast, cysteine and cysteinamide did not show any cytoprotective effects up to 4.0 mM, while exhibiting significant cytotoxic effects at high concentrations (at 2.0 mM and/or 4.0 mM). EDTA, a reference compound, did not show any cytoprotective effects up to 4.0 mM, while decreasing cell viability in a dose-dependent manner starting from a concentration as low as 0.25 mM.

### 3.5. Effects of Histidine, Histidinamide, Cysteine, and Cysteinamide on the CuSO_4_-Induced ROS production in HaCaT Cells

**Figure 5:**
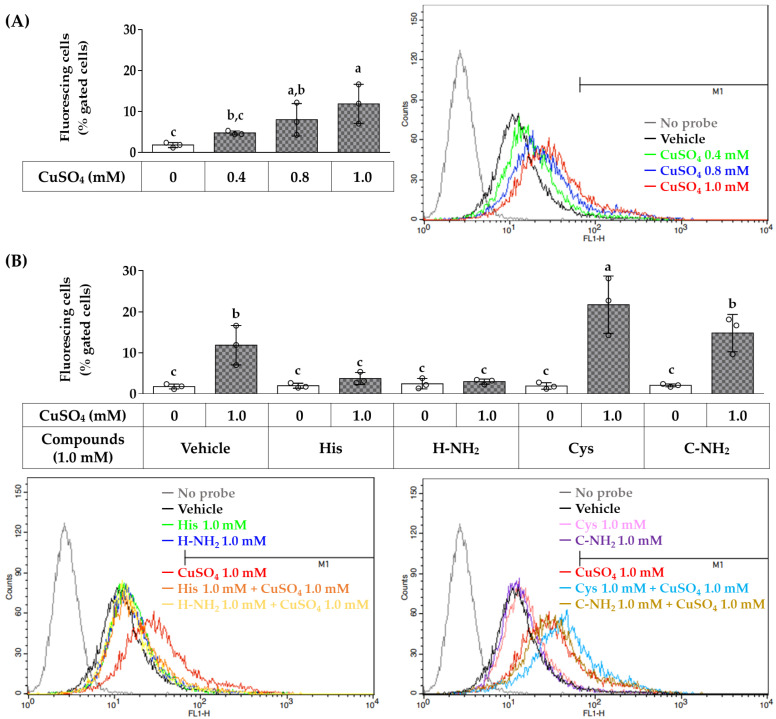
Flow cytometry for the assay of intracellular reactive oxygen species (ROS) production in HaCaT cells exposed to CuSO_4_ in the absence and presence of histidine, histidinamide, cysteine, or cysteinamide. In (**A**), cells were treated with CuSO_4_ at different concentrations for 3 h. In (**B**), cells were treated with vehicle or amino acid analog at 1.0 mM alone or in combination with 1.0 mM CuSO_4_ for 3 h. The treated cells were washed with phosphate-buffered saline (PBS), labeled with 2′,7′-dichlorodihydrofluorescein diacetate (DCFH-DA) for 30 min, and suspended in PBS for flow cytometric analysis. Typical flow cytometry histograms are shown. M1 in the histogram denotes the fluorescing cells. The ratios (%) of fluorescing cells to the total gated cells are presented (mean ± SD, n = 3). Different lowercase letters (a–c) indicate significantly different means at the *p* < 0.05 level.

The effects of histidine, histidinamide, cysteine, and cysteinamide on cellular ROS production were examined in the absence and presence of CuSO_4_ exposure. After various treatments of cells with CuSO_4_ and/or amino acids for 3 h, cells were labeled with a redox-sensitive probe (DCFH-DA), and the population of cells fluorescing due to ROS production was analyzed using flow cytometry. As shown in Figure 5, CuSO_4_ increased intracellular ROS production in a concentration-dependent manner. Although histidine and histidinamide did not affect basal ROS production, they inhibited the CuSO_4_-induced ROS production significantly. Cysteine did not affect the basal ROS production but rather enhanced the CuSO_4_-induced ROS production. Cysteinamide did not affect the basal or CuSO_4_-induced ROS productions.

### 3.6. Effects of Histidine, Histidinamide, Cysteine, and Cysteinamide on the GSH and GSSG Levels in HaCaT Cells Exposed to CuSO_4_

**Figure 6:**
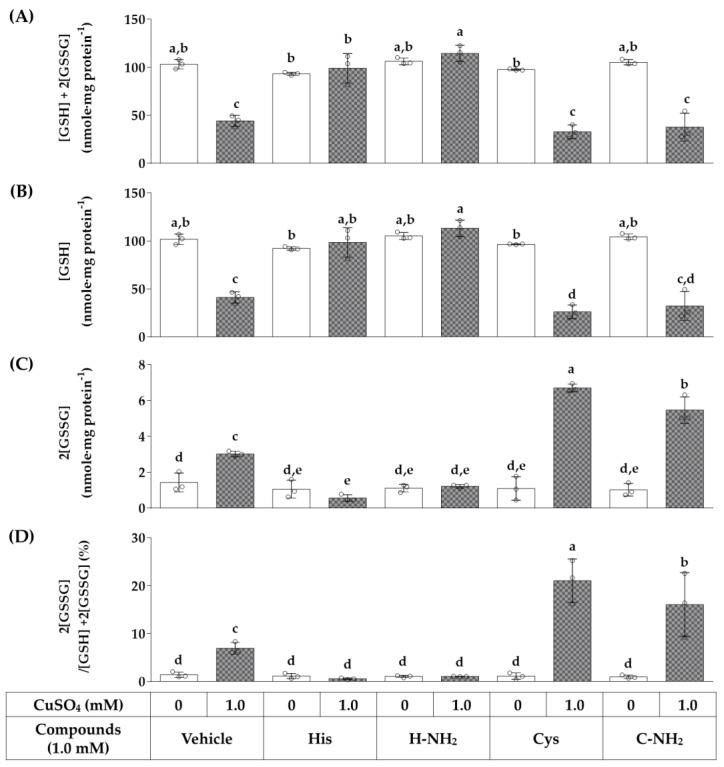
Effects of histidine, histidinamide, cysteine, and cysteinamide on the contents of glutathione (GSH) and glutathione disulfide (GSSG) in HaCaT cells exposed to CuSO_4_. Cells were treated with each compound at 1.0 mM in the absence and presence of 1.0 mM CuSO_4_ for 12 h. The total content of GSH plus GSSG (**A**) and that of GSH (**B**) were measured separately using an enzyme-based recycling assay, and the values were used to calculate the content of GSSG (**C**) and the ratio of the oxidized and reduced forms (**D**). Data are presented as mean ± SD (n = 3). Different lowercase letters (a–e) indicate significantly different means at the *p* < 0.05 level.

The next experiment examined the effects of histidine, histidinamide, cysteine, and cysteinamide on the cellular levels of GSH and its oxidized form, GSSG, in the absence and presence of CuSO_4_ exposure. As shown in Figure 6, histidine, histidinamide, cysteine, and cysteinamide did not affect the contents of GSH and GSSG, their total contents, or their relative ratio in the absence of CuSO_4_ exposure. As expected, CuSO_4_ exposure decreased GSH content and increased GSSG content, resulting in a decrease in their sum and an increase in the ratio of the oxidized form to the reduced form. In the presence of CuSO_4_ exposure, histidine and histidinamide mitigated the decrease in the total GSH pool and the increase in oxidized form caused by CuSO_4_ exposure, whereas cysteine and cysteinamide amplified these changes.

### 3.7. Effects of Histidine, Histidinamide, Cysteine, and Cysteinamide on the CuSO_4_-Induced Lipid Peroxidation and Protein Carbonylation in HaCaT Cells

**Figure 7:**
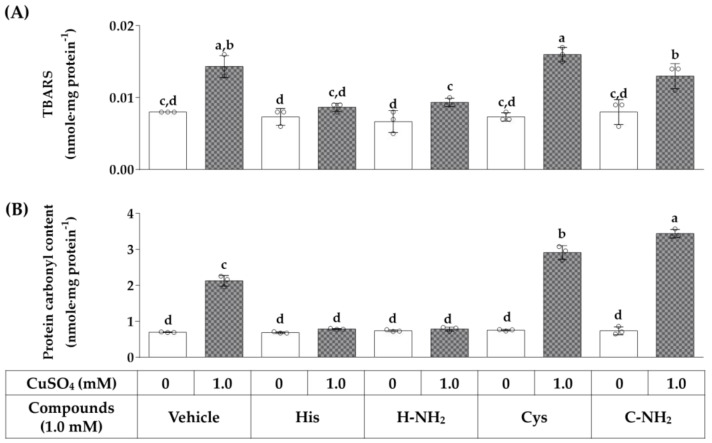
Effects of histidine, histidinamide, cysteine, and cysteinamide on the lipid peroxidation (**A**) and protein carbonylation (**B**) in HaCaT cells exposed to CuSO_4_. Cells were treated with each compound at 1.0 mM in the absence and presence of 1.0 mM CuSO_4_ for 12 h. The content of thiobarbituric acid-reactive substance (TBARS) as an indication of lipid peroxidation and protein carbonyl content as an indication of protein oxidation were normalized to the protein content. Data represent mean ± SD (n = 3). Different lowercase letters (a–d) indicate significantly different means at the *p* < 0.05 level.

We additionally examined the effects of histidine, histidinamide, cysteine, and cysteinamide on lipid peroxidation and protein carbonylation in HaCaT cells exposed to CuSO_4_. As shown in Figure 7, histidine, histidinamide, cysteine, and cysteinamide did not affect lipid peroxidation in cells under basal conditions. CuSO_4_ treatment significantly increased lipid peroxidation and the change was abrogated by histidine and histidinamide, whereas cysteine and cysteinamide had no significant effects. Histidine, histidinamide, cysteine, and cysteinamide had no effects on the basal levels of protein carbonylation in cells. CuSO_4_ treatment markedly increased protein carbonylation, and this change was abrogated by histidine and histidinamide. Cysteine and cysteinamide rather enhanced the protein carbonylation induced by CuSO_4_.

### 3.8. Comparison of Copper-Chelating Activities of Cysteine, Cysteinamide, Histidine, Histidinamide, and BSA

**Figure 8:**
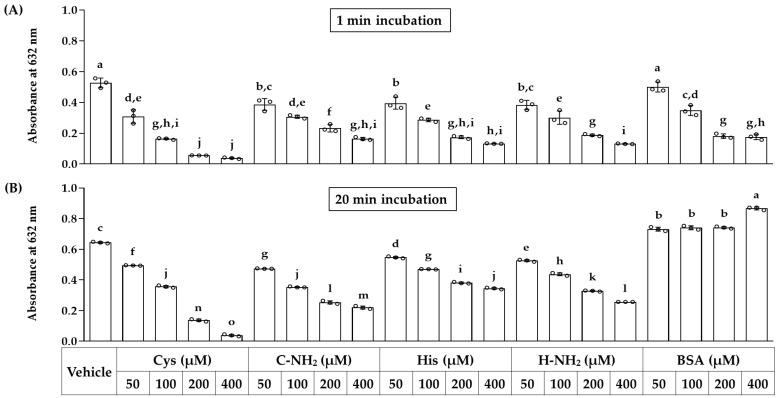
Comparison of copper-chelating activities of cysteine, cysteinamide, histidine, histidinamide, and bovine serum albumin (BSA). The reaction mixture consisting of 200 μM CuSO_4_, 200 μM PCV, and test material at the specified concentrations was incubated for 1 min (**A**) or 20 min (**B**), and the formation of Cu(II)-PCV complex was measured using A_632_. Data are presented as mean ± SD (n = 3). Different lowercase letters (a–o) indicate significantly different means at the *p* < 0.05 level.

Serum albumin is a macromolecule with a very large molecular weight (MW) that is involved in binding and transporting copper ions in the blood. In the following experiment, the copper chelation activity of BSA (MW 68,000) was compared with that of cysteine (MW 121), cysteinamide (MW 120), histidine (MW 155), and histidinamide (MW 154) at the same molar concentration ranges (50–400 µM). In the colorimetric assay monitoring A_632_ due to the formation of Cu(II)-PCV complex, the reaction mixtures were incubated for either 1 min or 20 min, and the results are shown in Figure 8. The results from the 1 min reaction showed that BSA reduced A_632_ to a similar extent as exhibited by cysteine, cysteinamide, histidine, and histidinamide at the same molar concentrations, even though its MW is 500 times greater than the amino acids. The result of the 20 min reaction indicated that BSA rather increased A_632_ due to the slow formation of a purple chromogen [37]. Thus, the results of the 1 min reaction are more likely to represent the copper chelation activity of BSA.

### 3.9. Effects of Cysteine, Cysteinamide, Histidine, Histidinamide, and BSA on the Viabilities of HaCaT Cells Exposed to CuSO_4_ or CuCl_2_

**Figure 9:**
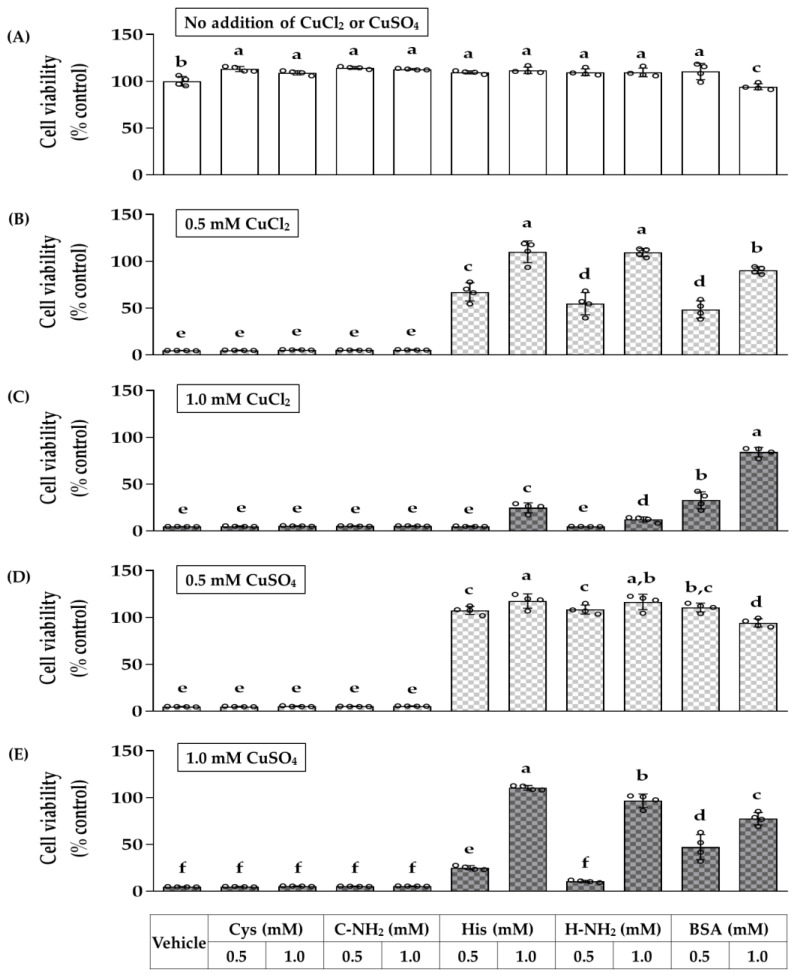
Effects of cysteine, cysteinamide, histidine, histidinamide, and BSA on the viability of HaCaT cells exposed to different Cu(II) salts. Cells were treated with a test material at varying concentrations with no addition (**A**) or the addition of 0.5 mM CuCl_2_ (**B**), 1.0 mM CuCl_2_ (**C**), 0.5 mM CuSO_4_ (**D**), or 1.0 mM CuSO_4_ (**E**) for 48 h. Cell viability is presented as % of the control group (mean ± SD, n = 4). Different lowercase letters (a–f) indicate significantly different means at the *p* < 0.05 level.

In the following experiments, the effects of cysteine, cysteinamide, histidine, histidinamide, and BSA on the viability of cells exposed to different types of Cu(II) salts were investigated. As shown in Figure 9, both CuCl_2_ and CuSO_4_ induced a high rate of cell death at 0.5 mM and 1.0 mM. Cysteine and cysteinamide did not affect the cytotoxic effects of CuCl_2_ and CuSO_4_. The viability of the cells exposed to 0.5 mM CuCl_2_ was recovered by half by 0.5 mM of histidine or histidinamide, and almost fully recovered by 1.0 mM of histidine or histidinamide. However, the viability of the cells exposed to 1.0 mM CuCl_2_ was restored slightly by 1.0 mM of histidine or histidinamide. The viability of the cells exposed to 0.5 mM CuSO_4_ was almost recovered by 0.5 mM and 1.0 mM of histidine or histidinamide. The viability of the cells exposed to 1.0 mM CuSO_4_ was almost recovered by 1.0 mM of histidine or histidinamide, but only partially recovered by 0.5 mM of histidine or histidinamide. BSA recovered about half the viability of cells exposed to 0.5–1.0 mM CuCl_2_ at 0.5 mM, and most recovered at 1 mM. BSA almost recovered the viability of the cells exposed to 0.5 mM CuSO_4_ at 0.5–1.0 mM, and recovered the viability of the cells exposed to 1.0 mM CuSO_4_ partly in a dose-dependent manner. Overall, the toxicity of Cu(II) ion was observed, regardless of the salt form. The protective effects of histidine and histidinamide against the toxicity of Cu(II) ion in cells were different depending on the concentrations and types of copper(II) salts. The cytoprotective effects of histidine and histidinamide were as powerful as BSA protein of the same molar concentration despite large differences in MW.

## 4. Discussion

Although previous studies have shown that copper chelators can efficiently remove Cu(II) ions from Cu(II) ion-overloaded cells and mitigate oxidative stress [38], the present study comprehensively compared the copper-chelating action of various amino acids and their action of alleviating copper ion-induced cytotoxicity for the first time, and several important novel findings were made. Despite the strongest copper chelation activities of cysteine and cysteinamide, they did not show any cytoprotective effects against CuSO_4_. In contrast, histidine and histidinamide prevented CuSO_4_-induced cell death although their copper-chelating activities were not stronger than that of cysteine or cysteinamide. The results support our hypothesis that only certain types of amino acids with copper chelation activity may have beneficial effects in cells exposed to high levels of copper ions.

Cu(II) ion is reduced to Cu(I) ion by reacting with biological molecules, such as ascorbic acid and GSH, or ROS, such as superoxide (O_2_^●−^) and hydrogen peroxide (H_2_O_2_) [39]. The resulting Cu(I) ion reacts with H_2_O_2_ through a Fenton-type reaction to generate a hydroxide ion (OH^−^) and hydroxyl radical (HO^●^) [40]. The generated Cu(II) ion is reduced back to Cu(I) ion to continue the cycle. When the Cu(II) ion is reduced by O_2_^●−^, this cycle constitutes a Haber–Weiss reaction [41]. Various metal chelators have differential effects on the generation of HO^●^ by promoting or inhibiting the Fenton-type reaction depending on the reaction conditions [25]. In the present study, various amino acids showed different copper-chelating activities, and their effects on the viability of Cu(II) ion-overloaded cells were partly associated with copper-chelating activities. The copper-chelating property of histidine and histidinamide is considered one of the contributing factors to their cytoprotective and antioxidant effects.

Cellular oxidative stress manifests as an increase in the ratio of prooxidants to antioxidants and an increase in the oxidative damage of cellular components [42,43]. For example, atmospheric particulate matter increases cellular ROS production, oxidizes GSH antioxidants, and increases lipid peroxidation, protein carbonylation, and DNA damage [33,34,35]. In previous studies, copper ions increased ROS production, lipid peroxidation, protein oxidation, and DNA damage in cells while decreasing the levels of small molecule antioxidants and antioxidant enzymes and inducing apoptosis and autophagy [17,18,44]. In the present study, CuSO_4_ dose-dependently increased cell death in the range of 0.2–1.0 mM. CuSO_4_ also increased cellular ROS production, GSH oxidation, lipid peroxidation, and protein carbonylation in cells. Therefore, the cytotoxicity of CuSO_4_ is closely related to oxidative stress. In addition to inducing oxidative stress, a high concentration of copper ions can displace other essential metal factors bound to macromolecules, negatively affecting the metabolism, gene expression, and viability of cells [12].

Copper ions are a type of Lewis acid and can bind with Lewis bases, which provide electron pairs for the formation of Lewis adducts. Copper ions are bonded via the sulfur atom of the thiol group of cysteine, the nitrogen atom of the imidazole ring of histidine, and the oxygen atom of the carboxyl group of the side chains of glutamic acid and aspartic acid [45]. In this study, cysteine and cysteinamide showed stronger copper-binding strength than histidine and histidinamide, respectively. We initially assumed that the negatively charged carboxyl group of free amino acids and the uncharged amide group of amidated amino acids might have different contributions to the copper ion-binding capacity. However, the differences in copper-chelating activity between histidine and histidinamide, or between cysteine and cysteinamide, were not consistently observed. In addition, the differences in cytoprotective effect between histidine and histidinamide, or between cysteine and cysteinamide, were not clear either. Thus, we tentatively concluded that the amide nitrogen atoms of the amidated amino acids have minor effects on the copper-binding activity or the cytoprotective effect.

EDTA, one of the typical metal chelators, has been shown to mitigate copper-induced toxicity in animal and plant models [46,47]. However, EDTA did not reduce protein carbonylation in THP-1 cells exposed to tobacco smoke extract, whereas another copper(II) ion chelator, d-penicillamine, did [48]. Thus, the biological effects of EDTA can vary under different conditions. In the present study, EDTA showed the highest copper-chelating activity among the tested compounds but did not prevent cell death induced by copper ions. Additionally, EDTA itself decreased cell viability in a concentration-dependent manner at concentrations above 0.25 mM in the absence of external copper ions. Although the precise mechanism for its toxic effects is currently unknown, it is probable that high concentrations of EDTA disrupt cellular metal homeostasis due to its potent and broad-spectrum metal chelation activity. EDTA also can exert oxidative stress in cells under certain conditions. In support of this notion, EDTA enhanced the oxidations of methyl linoleate induced by Fe(III) ion, whereas it suppressed the Cu(II) ion-induced oxidation [49].

GHK tripeptide is a copper carrier naturally found in plasma, saliva, and urine, and used in topical applications for skin regeneration purposes [50]. In the present study, the GHK-Cu complex did not affect the formation of the Cu(II)-PCV complex and the CuSO_4_-induced cytotoxicity, likely because the tripeptide is already saturated with copper ions.

The cytoprotective action of histidine and histidinamide against copper ions is considered very unique. Histidine and histidinamide were stronger than cysteine and cysteinamide in mitigating CuSO_4_-induced oxidative stress and enhancing cell survival. Cell death induced by 1.0 mM CuSO_4_ was almost completely blocked by the equivalent concentration of histidine or histidinamide. In addition, histidine and histidinamide at 1.0 mM suppressed cellular ROS production, GSH oxidation, lipid peroxidation, and protein carbonylation induced by 1.0 mM CuSO_4_. Thus, it is suggested that histidine or histidinamide may form a stable complex with copper ions and keep the concentration of catalytic copper ions very low, preventing ROS production.

In the present study, we further compared the copper-chelating activities of cysteine, cysteinamide, histidine, and histidinamide with that of BSA protein. BSA was tested as a representative copper-binding macromolecule because it is present in blood at a high concentration and is commercially available. We also compared the effects of cysteine, cysteinamide, histidine, histidinamide, and BSA on the viability of HaCaT cells exposed to different salts of Cu(II).

Normally, adult blood contains 35-50 mg mL^−1^ (0.51–0.74 mM) albumin [51], 71 μM histidine [52], and 16.7 μM copper ions [53]. Histidine and histidinamide have a MW of about 1/500 of BSA. In the present study, histidine and histidinamide exhibited copper chelation and copper cytotoxicity reduction as potent as BSA of the same molar concentration, indicating that the former compounds can have equivalent effects even with 1/500 the amount of BSA. In addition, histidine and histidinamide showed cytoprotective effects against copper toxicity at concentrations 1–2 times the concentration of copper ions. Therefore, the target concentration of histidine and histidinamide can be chosen in the range of 1–2 times the copper concentration in the skin or other body tissues determined in advance.

## 5. Conclusions

The results of this study suggest that histidine and histidinamide have very advantageous properties over cysteine and cysteinamide in terms of alleviating the oxidative stress and death of cells induced by copper ions. Further studies in vivo and clinical trials are necessary to examine the applicability of histidine and histidinamide to the treatment of diseases of the skin and other organs induced by high concentrations of copper ions.

## Data Availability

Not applicable.

## References

[1] Chauhan G., Pant K.K., Nigam K.D.P. (2015). Chelation technology: A promising green approach for resource management and waste minimization. Environ. Sci. Process. Impacts.

[2] Pinto I.S.S., Neto I.F.F., Soares H.M.V.M. (2014). Biodegradable chelating agents for industrial, domestic, and agricultural applications-a review. Environ. Sci. Pollut. Res..

[3] Franz K.J. (2013). Clawing back: Broadening the notion of metal chelators in medicine. Curr. Opin. Chem. Biol..

[4] Hu A.H., Wilson J.J. (2022). Advancing Chelation Strategies for Large Metal Ions for Nuclear Medicine Applications. Acc. Chem. Res..

[5] Tegoni M., Valensin D., Toso L., Remelli M. (2014). Copper Chelators: Chemical Properties and Bio-medical Applications. Curr. Med. Chem..

[6] Kontoghiorghes G.J., Kontoghiorghe C.N. (2020). Iron and Chelation in Biochemistry and Medicine: New Approaches to Controlling Iron Metabolism and Treating Related Diseases. Cells.

[7] Tapiero H., Townsend D.M., Tew K.D. (2003). Trace elements in human physiology and pathology. Copper. Biomed. Pharmacother..

[8] Tishchenko K.I., Beloglazkina E.K., Mazhuga A.G., Zyk N.V. (2016). Copper-containing enzymes: Site types and low-molecular-weight model compounds. Rev. J. Chem..

[9] Wazir S.M., Ghobrial I. (2017). Copper deficiency, a new triad: Anemia, leucopenia, and myeloneuropathy. J. Community Hosp. Intern. Med. Perspect..

[10] Taylor A.A., Tsuji J.S., Garry M.R., McArdle M.E., Goodfellow W.L., Adams W.J., Menzie C.A. (2020). Critical Review of Exposure and Effects: Implications for Setting Regulatory Health Criteria for Ingested Copper. Environ. Manag..

[11] Stern B.R. (2010). Essentiality and Toxicity in Copper Health Risk Assessment: Overview, Update and Regulatory Considerations. J. Toxicol. Environ. Health-Part A-Curr. Issues.

[12] Gaetke L.M., Chow-Johnson H.S., Chow C.K. (2014). Copper: Toxicological relevance and mechanisms. Arch. Toxicol..

[13] Twomey P.J., Vijoen A., House I.M., Reynolds T.M., Wierzbicki A.S. (2005). Relationship between serum copper, ceruloplasmin, and non-ceruloplasmin-bound copper in routine clinical practice. Clin. Chem..

[14] Pham A.N., Xing G.W., Miller C.J., Waite T.D. (2013). Fenton-like copper redox chemistry revisited: Hydrogen peroxide and superoxide mediation of copper-catalyzed oxidant production. J. Catal..

[15] Li H.R., Toh P.Z., Tan J.Y., Zin M.T., Lee C.Y., Li B., Leolukman M., Bao H.Q., Kang L.F. (2016). Selected Biomarkers Revealed Potential Skin Toxicity Caused by Certain Copper Compounds. Sci. Rep..

[16] Shaligram S., Campbell A. (2013). Toxicity of copper salts is dependent on solubility profile and cell type tested. Toxicol. Vitr..

[17] Husain N., Mahmood R. (2019). Copper(II) generates ROS and RNS, impairs antioxidant system and damages membrane and DNA in human blood cells. Environ. Sci. Pollut. Res..

[18] Kang Z.L., Qiao N., Liu G.Y., Chen H.M., Tang Z.X., Li Y. (2019). Copper-induced apoptosis and autophagy through oxidative stress-mediated mitochondrial dysfunction in male germ cells. Toxicol. Vitr..

[19] Baldari S., Di Rocco G., Toietta G. (2020). Current Biomedical Use of Copper Chelation Therapy. Int. J. Mol. Sci..

[20] Tripathi I.P., Kamal A. (2015). Synthesis, Characterization of Some Complexes of Copper (II) with L-Asparginine, L-Histidine, L-Lysine. Am. J. Adv. Drug Deliv..

[21] Deschamps P., Kulkarni P.P., Gautam-Basak M., Sarkar B. (2005). The saga of copper(II)-L-histidine. Coord. Chem. Rev..

[22] Sheela S.R., Latha M., Liu P., Lem K., Kaler S.G. (2005). Copper-replacement treatment for symptomatic Menkes disease: Ethical considerations. Clin. Genet..

[23] Imer F., Aldemir E., Kilic H., Sonmezoglu I., Apak R. (2008). The Protective Effect of Amino Acids on the Copper(II)-Catalyzed Autoxidation of Ascorbic Acid. J. Food Drug Anal..

[24] Jiang W.D., Qu B., Feng L., Jiang J., Kuang S.Y., Wu P., Tang L., Tang W.N., Zhang Y.A., Zhou X.Q. (2016). Histidine Prevents Cu-Induced Oxidative Stress and the Associated Decreases in mRNA from Encoding Tight Junction Proteins in the Intestine of Grass Carp (*Ctenopharyngodon idella*). PLoS ONE.

[25] Timoshnikov V.A., Selyutina O.Y., Polyakov N.E., Didichenko V., Kontoghiorghes G.J. (2022). Mechanistic Insights of Chelator Complexes with Essential Transition Metals: Antioxidant/Pro-Oxidant Activity and Applications in Medicine. Int. J. Mol. Sci..

[26] Kim J.H., Seok J.K., Kim Y.M., Boo Y.C. (2019). Identification of small peptides and glycinamide that inhibit melanin synthesis using a positional scanning synthetic peptide combinatorial library. Br. J. Derm..

[27] Lee H.K., Ha J.W., Hwang Y.J., Boo Y.C. (2021). Identification of L-Cysteinamide as a Potent Inhibitor of Tyrosinase-Mediated Dopachrome Formation and Eumelanin Synthesis. Antioxidants.

[28] Lee J.E., Boo Y.C. (2022). Combination of Glycinamide and Ascorbic Acid Synergistically Promotes Collagen Production and Wound Healing in Human Dermal Fibroblasts. Biomedicines.

[29] Okajima S., Hamamoto A., Asano M., Isogawa K., Ito H., Kato S., Hirata Y., Furuta K., Takemori H. (2019). Azepine derivative T4FAT, a new copper chelator, inhibits tyrosinase. Biochem. Biophys. Res. Commun..

[30] Stockert J.C., Horobin R.W., Colombo L.L., Blazquez-Castro A. (2018). Tetrazolium salts and formazan products in Cell Biology: Viability assessment, fluorescence imaging, and labeling perspectives. Acta Histochem..

[31] Eruslanov E., Kusmartsev S. (2010). Identification of ROS using oxidized DCFDA and flow-cytometry. Methods Mol. Biol..

[32] Lai W.W., Hsiao Y.P., Chung J.G., Wei Y.H., Cheng Y.W., Yang J.H. (2011). Synergistic phototoxic effects of glycolic acid in a human keratinocyte cell line (HaCaT). J. Derm. Sci..

[33] Piao M.J., Ahn M.J., Kang K.A., Ryu Y.S., Hyun Y.J., Shilnikova K., Zhen A.X., Jeong J.W., Choi Y.H., Kang H.K. (2018). Particulate matter 2.5 damages skin cells by inducing oxidative stress, subcellular organelle dysfunction, and apoptosis. Arch. Toxicol..

[34] Ha J.W., Boo Y.C. (2021). Siegesbeckiae Herba Extract and Chlorogenic Acid Ameliorate the Death of HaCaT Keratinocytes Exposed to Airborne Particulate Matter by Mitigating Oxidative Stress. Antioxidants.

[35] Bae I.A., Ha J.W., Choi J.Y., Boo Y.C. (2022). Antioxidant Effects of Korean Propolis in HaCaT Keratinocytes Exposed to Particulate Matter 10. Antioxidants.

[36] Ghani M.A., Barril C., Bedgood D.R., Prenzler P.D. (2017). Measurement of antioxidant activity with the thiobarbituric acid reactive substances assay. Food Chem..

[37] Huang T., Long M., Huo B. (2010). Competitive Binding to Cuprous Ions of Protein and BCA in the Bicinchoninic Acid Protein Assay. Open Biomed. Eng. J..

[38] Rakshit A., Khatua K., Shanbhag V., Comba P., Datta A. (2018). Cu(2+) selective chelators relieve copper-induced oxidative stress in vivo. Chem. Sci..

[39] Ohta Y., Shiraishi N., Nishikawa T., Nishikimi M. (2000). Copper-catalyzed autoxidations of GSH and L-ascorbic acid: Mutual inhibition of the respective oxidations by their coexistence. Biochim. Biophys. Acta-Gen. Subj..

[40] Timoshnikov V.A., Kobzeva T., Selyutina O.Y., Polyakov N.E., Kontoghiorghes G.J. (2019). Effective inhibition of copper-catalyzed production of hydroxyl radicals by deferiprone. J. Biol. Inorg. Chem..

[41] Kehrer J.P. (2000). The Haber-Weiss reaction and mechanisms of toxicity. Toxicology.

[42] Akagawa M. (2021). Protein carbonylation: Molecular mechanisms, biological implications, and analytical approaches. Free Radic. Res..

[43] Pizzino G., Irrera N., Cucinotta M., Pallio G., Mannino F., Arcoraci V., Squadrito F., Altavilla D., Bitto A. (2017). Oxidative Stress: Harms and Benefits for Human Health. Oxid. Med. Cell. Longev..

[44] Yang F., Pei R.N., Zhang Z.W., Liao J.Z., Yu W.L., Qiao N., Han Q.Y., Li Y., Hu L.M., Guo J.Y. (2019). Copper induces oxidative stress and apoptosis through mitochondria-mediated pathway in chicken hepatocytes. Toxicol. Vitr..

[45] Lawson M.K., Valko M., Cronin M.T.D., Jomová K. (2016). Chelators in Iron and Copper Toxicity. Curr. Pharmacol. Rep..

[46] Harrington J.M., Boyd W.A., Smith M.V., Rice J.R., Freedman J.H., Crumbliss A.L. (2012). Amelioration of Metal-Induced Toxicity in Caenorhabditis elegans: Utility of Chelating Agents in the Bioremediation of Metals. Toxicol. Sci..

[47] Saleem M.H., Ali S., Kamran M., Iqbal N., Azeem M., Javed M.T., Ali Q., Haider M.Z., Irshad S., Rizwan M. (2020). Ethylenediaminetetraacetic Acid (EDTA) Mitigates the Toxic Effect of Excessive Copper Concentrations on Growth, Gaseous Exchange and Chloroplast Ultrastructure of Corchorus capsularis L. and Improves Copper Accumulation Capabilities. Plants.

[48] Lin C.C., Su T.H., Wang T.S. (2009). Protein carbonylation in THP-1 cells induced by cigarette smoke extract via a copper-catalyzed pathway. Chem. Res. Toxicol..

[49] Yoshida Y., Furuta S., Niki E. (1993). Effects of metal chelating agents on the oxidation of lipids induced by copper and iron. Biochim. Biophys. Acta.

[50] Pickart L., Vasquez-Soltero J.M., Margolina A. (2015). GHK Peptide as a Natural Modulator of Multiple Cellular Pathways in Skin Regeneration. Biomed Res. Int..

[51] Sheinenzon A., Shehadeh M., Michelis R., Shaoul E., Ronen O. (2021). Serum albumin levels and inflammation. Int. J. Biol. Macromol..

[52] Hortin G.L., Landt M., Powderly W.G. (1994). Changes in plasma amino acid concentrations in response to HIV-1 infection. Clin. Chem..

[53] Kirsipuu T., Zadoroznaja A., Smirnova J., Friedemann M., Plitz T., Tougu V., Palumaa P. (2020). Copper(II)-binding equilibria in human blood. Sci. Rep..

